# *lemmingA *encodes the Apc11 subunit of the APC/C in *Drosophila melanogaster *that forms a ternary complex with the E2-C type ubiquitin conjugating enzyme, Vihar and Morula/Apc2

**DOI:** 10.1186/1747-1028-7-9

**Published:** 2012-03-14

**Authors:** Olga Nagy, Margit Pál, Andor Udvardy, Christine AM Shirras, Imre Boros, Alan D Shirras, Péter Deák

**Affiliations:** 1 Institute of Biochemistry, Biological Research Centre, Szeged, Hungary; 2 Division of Biomedical and Life Sciences, Lancaster University, Lancaster, UK; 3 Department of Genetics, University of Szeged, Szeged, Hungary

## Abstract

**Background:**

Ubiquitin-dependent protein degradation is a critical step in key cell cycle events, such as metaphase-anaphase transition and mitotic exit. The anaphase promoting complex/cyclosome (APC/C) plays a pivotal role in these transitions by recognizing and marking regulatory proteins for proteasomal degradation. Its overall structure and function has been elucidated mostly in yeasts and mammalian cell lines. The APC/C is, however, a multisubunit assembly with at least 13 subunits and their function and interaction within the complex is still relatively uncharacterized, particularly in metazoan systems. Here, *lemming *(*lmg*) mutants were used to study the APC/C subunit, Apc11, and its interaction partners in *Drosophila melanogaster*.

**Results:**

The *lmg *gene was initially identified through a pharate adult lethal P element insertion mutation expressing developmental abnormalities and widespread apoptosis in larval imaginal discs and pupal abdominal histoblasts. Larval neuroblasts were observed to arrest mitosis in a metaphase-like state with highly condensed, scattered chromosomes and frequent polyploidy. These neuroblasts contain high levels of both cyclin A and cyclin B. The *lmg *gene was cloned by virtue of the *lmg^03424 ^*P element insertion which is located in the 5' untranslated region. The *lemming *locus is transcribed to give a 2.0 kb mRNA that contains two ORFs, *lmgA *and *lmgB*. The *lmgA *ORF codes for a putative protein with more than 80% sequence homology to the APC11 subunit of the human APC/C. The 85 amino acid protein also contains a RING-finger motif characteristic of known APC11 subunits. The *lmgA *ORF alone was sufficient to rescue the lethal and mitotic phenotypes of the *lmg^138 ^*null allele and to complement the temperature sensitive lethal phenotype of the *APC11-myc9 *budding yeast mutant. The LmgA protein interacts with Mr/Apc2, and they together form a binding site for Vihar, the E2-C type ubiquitin conjugating enzyme. Despite being conserved among *Drosophila *species, the LmgB protein is not required for viability or fertility.

**Conclusions:**

Our work provides insight into the subunit structure of the *Drosophila *APC/C with implications for its function. Based on the presented data, we suggest that the Lmg/Apc11 subunit recruits the E2-C type ubiquitin conjugating enzyme, Vihar, to the APC/C together with Mr/Apc2 by forming a ternary complex.

## Background

Chromosome separation at anaphase onset and exit from mitosis are regulated by ubiquitylation and subsequent degradation of key regulatory proteins, the securins and mitotic cyclins [1]. The ubiquitylation of these proteins is catalyzed by a cascade of E1, E2 and E3 enzymes, the crucial factor being the cell cycle regulated E3 ubiquitin protein ligase, the anaphase-promoting complex or cyclosome (APC/C) that provides the platform for the ubiquitylation reaction and determines substrate specificity.

The APC/C contains at least 13 different subunits in the budding yeast, *Saccharomyces cerevisiae*, and most of these subunits appear to be conserved in all eukaryotes with the exception of Apc9 to which no homologs have been identified in multicellular eukaryotes. In contrast, the Apc7 and Apc16 homologs have been identified in multicellular, but not in unicellular, eukaryotes [2,3].

An architectural analysis of the budding yeast APC/C revealed two subcomplexes that are held together by the largest subunit, Apc1 [4]. Apc1, together with subunits Apc4 and Apc5 serves as a scaffold for the whole complex. One of the subcomplexes connects through the Apc4 and Apc5 subunits and contains three subunits (Cdc16, Cdc23 and Cdc27), with tandem arrays of multiple tetratricopeptide repeats (TPR). Since the TPR motifs are generally involved in protein-protein interactions, they are thought to contribute to substrate binding. The other APC/C subcomplex contains Apc2, Apc10/Doc1 and Apc11 subunits, and connects to Apc1 through Apc2 [4]. It has been proposed that in yeast and in human cells, either the RING finger Apc11 subunit alone [5,6], or together with the cullin homolog Apc2 defines the minimal ubiquitin ligase activity of the APC/C, depending on the type of ubiquitin conjugating enzyme (E2) used in these reactions [7]. The gene encoding Apc11 has been cloned from the nematode, *Caenorhabditis elegans*. RNAi analysis in this species has shown that in the absence of APC11, zygotes arrested before the onset of cleavage. The absence of polar bodies in these zygotes indicates arrest during the first meiotic division of the oocyte [8]. In *Arabidopsis thaliana *APC11 interacts with APC2 and form a heterodimer complex [9]. In HeLa cells hydrogen peroxide induces zinc release from APC11, and impairs the interaction between APC11 and the E2 enzyme Ubc4 and therefore inhibits the ubiquitin ligase activity of APC11 [10]. The functions of other subunits remain obscure, though some of them, like Apc13 and Cdc26 were implicated in stabilizing the complex [11].

To understand the role of APC/C subunits in Drosophila, we previously undertook detailed genetic and molecular analyses of the genes coding for several subunits. We have shown that the Mákos/Cdc27, Cdc16 and Cdc23 TPR subunits and the Apc10/DOC1 subunit were essential and required to mediate progression through metaphase. The fourth TPR subunit, Apc7 is a nonessential component of the Drosophila APC/C with unknown function [12-14]. In this paper we present mutational and molecular analysis of the Drosophila *lemming *(*lmg*) gene and show that it has an unusual dicistronic mRNA. The upstream open reading frame (ORF), *lmgA *is an essential gene that encodes a functional homologue of the *S. cerevisiae *small APC/C subunit, Apc11. The downstream ORF, *lmgB *is dispensable without causing any detectable phenotype. Phenotypic analysis of hypomorphic and null *lmg *alleles revealed prometaphase cell cycle arrest and apoptosis, consistent with loss of APC/C function. Interacting partners of Lmg/Apc11 were also investigated by yeast two-hybrid analysis and their possible function will be discussed.

## Results

### Disruption of *lemming *results in pupal lethality and widespread apoptosis

**Figure 1:**
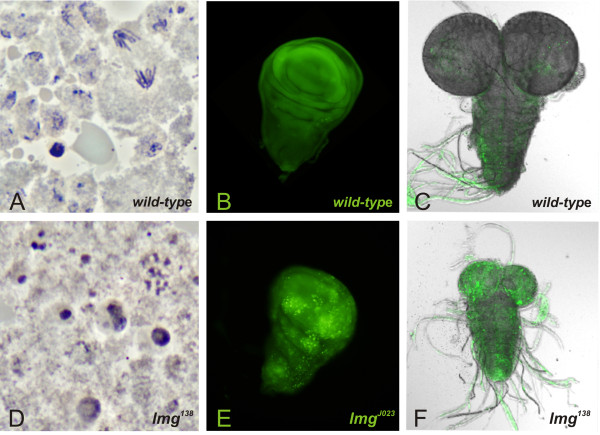
**Loss of *lmg *leads to elevated apoptosis in larval brain and imaginal discs**. Orcein-stained preparations of wild-type (A) and *lmg^138 ^*(D) larval brain squashes. *lmg *mutants show small, rounded cells with uneven nuclear staining. Acridine Orange staining also highlights the high incidence of dying cells in *lmg^J023 ^*mutant wing imaginal disc (E) and *lmg^138 ^*larval brain (F) relative to wild-type tissues (B and C). Green dots indicate apoptosis. The *lmg^138 ^*null mutant (F) has reduced brain and optic lobes compared to wild-type (C).

**Figure 2:**
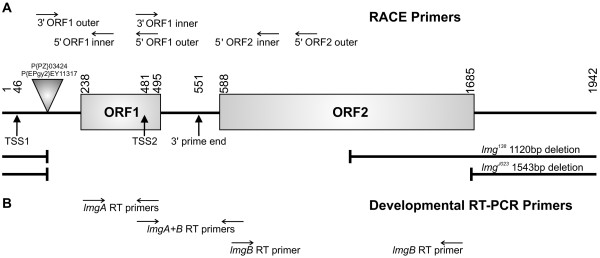
**Schematic representation of the *lmg *locus**. The molecular map of the *lmg *locus represents the ~2.0 kb cDNA sequence available in FlyBase (A). It indicates the two ORFs (rectangles), transcription start sites (TSS1 and 2), P element insertion sites (triangle), the extension of deletion in the *lmg^J023 ^*and *lmg^138 ^*alleles and the 3'-end of the monocistronic transcript identified by RACE. It also shows the relative location of RACE primers used in this work. The primer pairs used for developmental RT-PCR experiments and their relative locations are also displayed (B).

The *l(2)03424 *mutant stock was originally identified during a screen of the Berkeley *Drosophila *Genome Project (BDGP) collection of P element insertion lines for pharate adult lethal mutants with defective abdomens. *l(2)03424 *homozygotes die at pharate adult stage P15(i) (Table 1) [15] and additionally have reduced eyes and wings, as well as bristle defects. Acridine orange and TUNEL staining of imaginal discs showed that a likely cause of these defects was apoptosis of imaginal cells (data not shown). The gene containing the P element insertion was re-named *lemming *(*lmg*) and its first mutant allele was designated *lmg^03424^*. The P element was confirmed as the cause of the *lmg*^03424 ^mutation, as precise excisions reverted to a wild type phenotype. A stronger allele, *lmg^J023 ^*was established by imprecise excision of the P element by crossing to a Δ2-3 transposase-source strain. Most of the *lmg^J023 ^*homozygotes die as P5(i) stage pupae (Table 1). Orcein and acridine orange staining of larval brains and imaginal discs revealed widespread apoptosis (Figure 1). In addition, a non-complementing P element insertion allele, *lmg^EY11317^*, identified by the BDGP, showed P4(ii) lethality, and the imprecise excision of its P element yielded the strongest *lemming *allele, *lmg^138^*, with a lethal phase in P4(i) (Table 1). The *lmg^138 ^*mutation proved to represent a small deletion removing almost the entire coding region of the gene (Figure 2A); therefore it is considered a null allele of *lemming*. Dissected tissues showed that the optic lobes and imaginal discs of *lmg^138 ^*homozygotes were substantially reduced in size compared to wild type (Figure 1F).

**Table 1:** Quantitative analysis of lethal and mitotic phenotypes of *lmg *mutants

Genotype	Lethal phase	Preparations per fields	Mitotic index	M:A ratio	Polyploid %	Apoptotic index
**A**						

*w^11118^*	Viable	7/183	1.6 ± 0.2	2.9 ± 0.3	0	0.5 ± 0.1

*lmg^03424^*	Pharate adult P15(i)	6/135	4.1 ± 0.8	4.5 ± 0.7	19.6 ± 5.6	1.4 ± 0.3

*lmg^J023^*	Pupal stage P5(i)	7/167	4.4 ± 0.9	6.3 ± 0.1	29.1 ± 7.4	1.2 ± 0.2

*lmg^EY11317^*	Prepupal stage P4(ii)	6/170	5.8 ± 0.7	6.2 ± 0.4	26.6 ± 3.1	1.5 ± 0.4

*lmg^138^*	Prepupal stage P4(i)	10/194	5.9 ± 0.5	-	50.7 ± 7.9	1.8 ± 0.5

*lmg^138^;lmgA- pUAST/daGAL4*	Viable	6/180	1.5 ± 0.2	3 ± 0.6	0	0.5 ± 0.1

*lmg^138^;lmgA + B- pUAST/daGAL4*	Viable	4/100	1.4 ± 0.2	3.1 ± 0.3	0	0.6 ± 0.2

*lmg^138^;lmgB- pUAST/daGAL4*	Prepupal stage P4(i)	5/100	4.9 ± 0.9	-	43.7 ± 6.2	1.7 ± 0.2

**B**						

*mr^2^*	Female sterile	6/174	1.4 ± 0.1	3.3 ± 0.4	0	1.2 ± 0.3

*lmg^03424^, mr^2^*	Prepupal stage P4(ii)	6/106	4.5 ± 0.7	4.4 ± 0.3	31.8 ± 3.9	1.3 ± 0.4

**C**						

*vihar^S110501^*	Pharate adult P15(i)	7/196	2.1 ± 0.3	3.7 ± 0.6	4.2 ± 0.4	0.9 ± 0.3

*lmg^03424^, vihar^S110501^*	Bubble prepupa	6/116	6.1 ± 0.6	3.9 ± 0.7	44.3 ± 8.4	1.8 ± 0.3

	stage P3					

*mr^2^; vihar^S110501^*	Early L3 larvae	n.d.	n.d.	n.d.	n.d.	n.d.

*mr^2^; vihar^KG02013^*	Early L3 larvae	n.d.	n.d.	n.d.	n.d.	n.d.

The number of preparations and the total number of optical fields analyzed are given. Mitotic index is given as the average number of mitotic cells in an optical field (± sd). Polyploid% represents the percentage of mitotic cells (± sd) with higher than two sets of chromosomes. Apoptotic index is given as the mean number of apoptotic cells in a microscope field (± sd).

### *lemming *mutants show metaphase-like delay with overcondensed chromosomes

**Figure 3:**
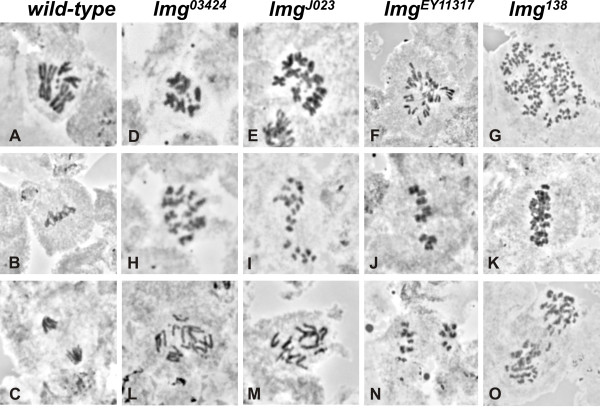
**Mitotic abnormalities of *lmg *alleles in orcein stained larval neuroblasts**. Wild-type mitotic cells in prometaphase (A), metaphase (B) and anaphase (C). Neuroblast cells from different *lmg *mutants show prometaphase- or metaphase-like arrest with overcondensed chromosomes and polyploidy (D-K). Chromosome overcondensation could also be observed in anaphase figures (N and O), together with scattered chromosome segregation (L and M). Many cells appear polyploid (G, K and O), and they invariantly have overcondensed chromosomes.

The apoptotic phenotype of *lmg *mutants is restricted to mitotically active cells suggesting that it may be caused by mitotic defects. We examined orcein-stained brain squash preparations for mitotic abnormalities from *lmg *mutant third instar larvae. Examples of mitotic figures in *lmg *neuroblasts are shown in Figure 3. Chromosomes appeared normal during prophase, but overcondensation was readily apparent at prometaphase and/or metaphase (Figure 3D-K). The proportion of cells in mitosis was much higher in the mutant preparations compared to wild type, most of the dividing cells being in a prometaphase- or metaphase-like stage. Cells in anaphase were rare and most of them appeared abnormal with highly condensed (Figure 3N and 3O), lagging chromosomes (Figure 3L and 3M). Polyploid cells with overcondensed chromosomes were frequently observed (~ in 30% of cells in mitosis; Figure 3G, K and 3O).

Larval brains of *lmg^03424^, lmg^EY11317 ^*and *lmg*^J023 ^had a high mitotic index (4.1 for *lmg*^03424^, 5.8 for *lmg^EY11317 ^*and 4.4 for *lmg*^J023^, n = 135, 170 and 167 respectively), compared to *w^1118 ^*(1.6), (Table 1). The high mitotic index and the extent of chromosome overcondensation are indicative of metaphase arrest. This is further supported by the high metaphase to anaphase ratios (4.5 for *lmg*^03424^, 6.2 for *lmg^EY11317 ^*and 6.3 for *lmg*^J023^). It should be mentioned that though anaphase still occurs at a lower rate in *lmg *mutants, true metaphase figures with chromosomes fully aligned to the metaphase plate were never observed, in spite of the fact, that such an alignment could be identified in wild type preparations in about 4-6% of mitotic cells. Instead, the cells persisted in a prometaphase- or metaphase-like state with scattered, highly condensed chromosomes.

### *lemming *mutants accumulate cyclin A and B

**Figure 4:**
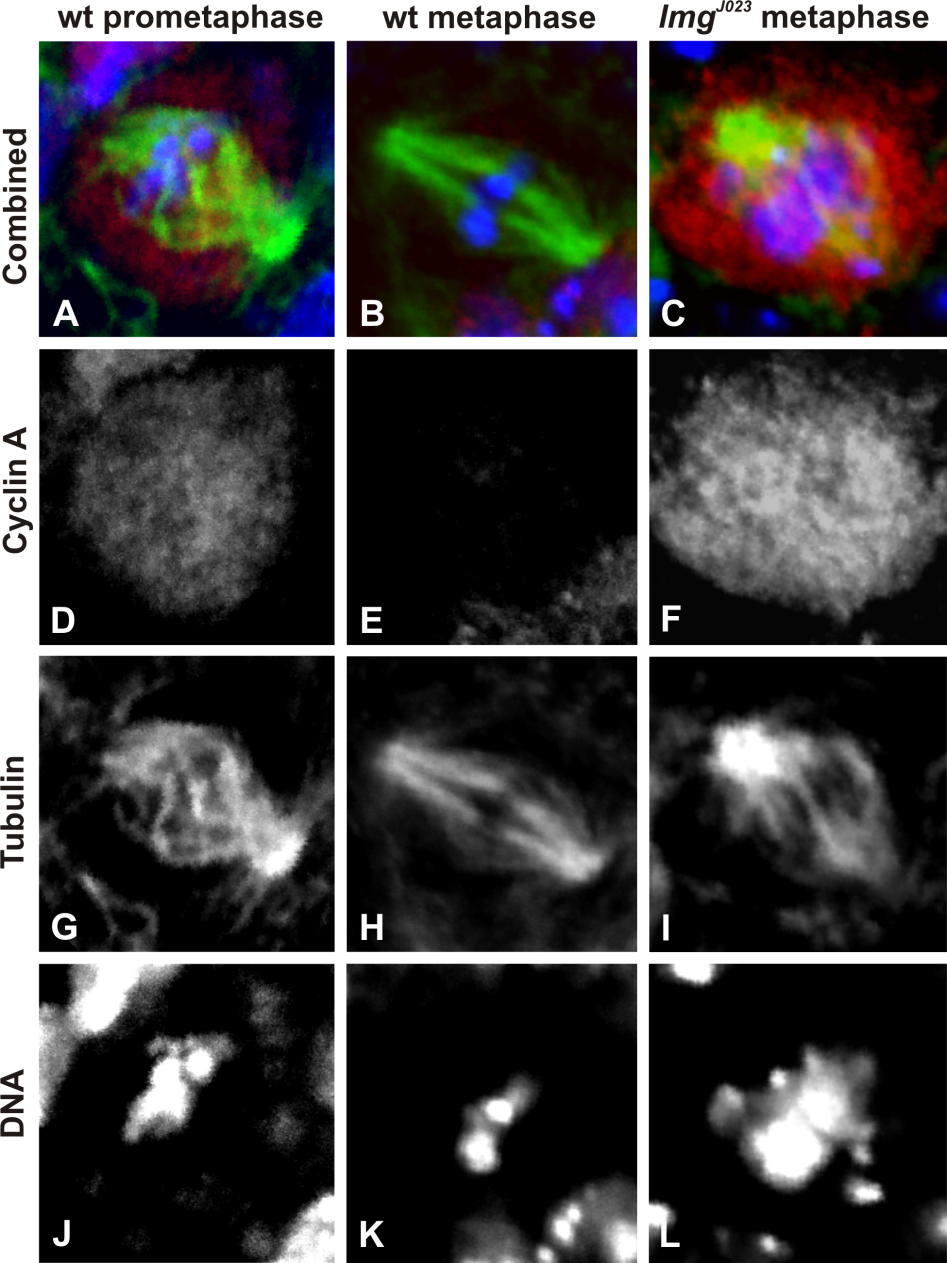
**Mitotic cyclinA is not degraded in *lmg *mutants**. Images show cyclin A (red in A, B, C, monochromatic in D, E, F), and tubulin (green in A, B, C, monochromatic in G, H, I) localizations as well as DNA staining (blue in A, B, C, monochromatic in J, K, L) in mitotic cells. In wild-type cells (*n *= 29) the cyclin A staining is visible in prophase or prometaphase (A and D), but undetectable in metaphase cells (B and E). In metaphase-like *lmg^J023 ^*cells (*n *= 17) cyclin A staining is intense (C and F).

**Figure 5:**
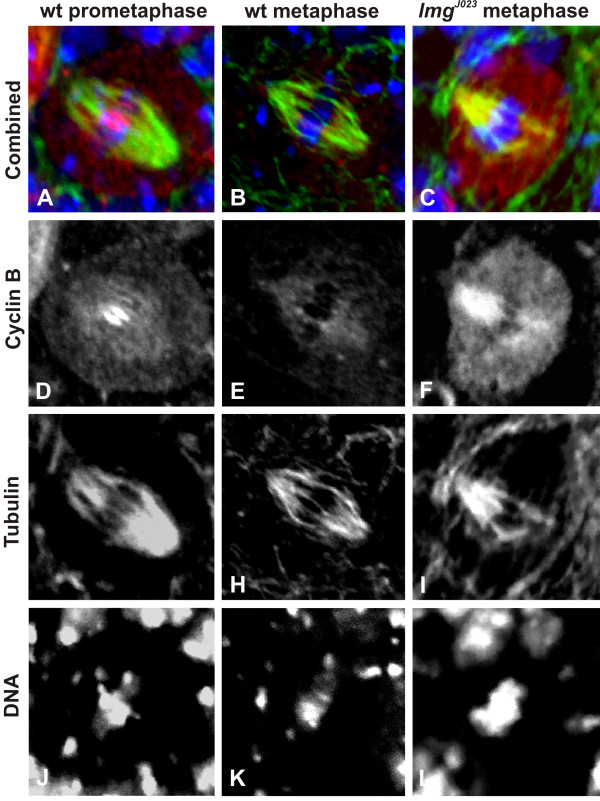
**CyclinB is not degraded in *lmg *mutants**. Colours are the same as in Figure 4. The level of Cyclin B is high in wild-type (*n *= 24) prophase and prometaphase cells (A and D) and it starts to disappear at or after the onset of metaphase (B and E). Cyclin B staining is quite pronounced in most of the *lmg^J023 ^*(C and F) cells (*n *= 20) arrested in metaphase.

Mitotic cyclins are one of the prime APC/C substrates. The APC/C targets the mitotic cyclins for degradation in late mitosis, thereby permitting mitotic exit [16]. We have shown previously, that *Apc3/Cdc27, Apc6/Cdc16 *and *Apc10 *mutants show elevated levels of cyclin A and cyclin B [12-14]. Therefore we tested if this function was affected in *lmg *mutants by monitoring cyclin levels in wild type and *lmg *mutant neuroblasts in immunostaining experiments with polyclonal antibodies against cyclin A and cyclin B. In wild type, a high level of cyclin A could be detected in prophase and early prometaphase cells (Figure 4A and 4D), identifiable on the basis of weaker and somewhat diffuse DNA signal, but it disappeared during metaphase (Figure 4B and 4E). Cyclin B was highly visible in prometaphase (Figure 5A and 5D), it started to disappear in metaphase (Figure 5B and 5E), and was undetectable in anaphase cells. Only 14% and 17% of cells with bright DNA staining retained cyclin A (n = 29) and cyclin B (n = 24) signals respectively. Both antisera gave strong staining in *lmg *mutant preparations as well. However, in contrast to wild type, more than 80% of *lmg^J023 ^*neuroblasts that showed intense DNA staining around the middle of the spindle, indicating arrest in a metaphase-like state, also showed visible accumulation of both cyclin A (n = 17; Figure 4C and 4F) and cyclin B (n = 20; Figure 5C and 5F), most abundantly around the mitotic spindle. This phenotype was similar in all *lmg *mutants, and indicates that the Lmg protein is required for cyclin A and cyclin B degradation.

### The *lemming *locus is dicistronic and its upstream ORF, *lmgA*, codes for the Apc11 subunit of the Drosophila APC/C

The P element insertions of *lmg^03424 ^*and *lmg^EY11317 ^*mutants were both mapped to the left arm of chromosome 2, to the cytogenetic location 29D4-5. Remobilization of these insertions resulted in frequent reversion of the mutant phenotype to wild type, suggesting that these insertions were directly responsible for disrupting gene function. Genomic DNA flanking the *lmg*^03424 ^insertion was recovered by plasmid rescue and used to probe larval cDNA libraries. Several overlapping clones were identified and their nucleotide sequences determined. BLAST searches of the Berkeley *Drosophila *Genome Project (BDGP) collection of ESTs revealed a number of embryonic cDNA clones which shared nucleotide sequence with the putative *lmg *cDNAs. One of these (LD20119) was completely sequenced and comparison with genomic sequence showed that the *lmg *locus is intronless. The 2.0 kb transcript contains two ORFs separated by 92 bases (Figure 2A). Sequence data of 423 cDNA clones for this locus are reported in FlyBase. They identify two cDNA classes based on size, a 2.0 and a 2.9 kb, both of which appear to be dicistronic. The 5'-ends of 230 cDNA clones identifies the transcription start site at 192 (± 4) bps upstream of the ORF1 start codon [17]. To better characterize the transcription profile of the *lemming *locus, RACE analyses were performed, using total RNA isolated from embryos and L3 larvae. Regardless of RNA source, the 5'-end of the transcript (using the ORF1 inner primer) could be placed at 180 (± 15) bases upstream of the ORF1 start. However, 5'-RACE analysis with the 5' ORF2 inner primer on embryonic RNA revealed two different 5'-ends: one extending 107 bases upstream of the ORF2 translation start site and the other one matching the 5'-end of ORF1 (Figure 2A). Similar 5'-RACE analysis (with the 5' ORF2 outer primer) on L3 larval RNA resulted in only one mRNA 5'-end that matched the 5'-end of embryonic mRNA upstream of ORF1, thus representing dicistronic mRNA. The 3' RACE experiments using ORF1 primers yielded an mRNA end located in the intercistronic region 56 bases downstream of the stop codon of ORF1. The determination of the 3'-end using ORF2 primers was ambiguous by 3' RACE, but sequencing the 3'-end of the cDNA clone identified in the yeast two hybrid experiments (see later) revealed the 3'-end at 1231 bases downstream of the stop codon closing ORF2. Based on this analysis, we concluded that the *lmg *locus gives rise to a bona fide dicistronic message and designated ORF1 and ORF2 as *lmgA *and *lmgB *respectively. The presence of monocistronic mRNAs could be explained by processing of the dicistronic messages, although it is still possible that original monocistronic mRNAs may be made.

The *lmgA *ORF encodes a small, putative polypeptide of 85 amino acids (~ 10 kDa), that contains a RING-finger motif characteristic of known APC11 subunits, and shows more than 80% sequence similarity with the APC11 subunit of the human APC/C. The *lmgB *ORF (corresponding to CG34441 in FlyBase) codes for a putative polypeptide of 365 amino acids, with no apparent functional domains. In a more detailed bioinformatic analysis of the *lemming *locus, the evolutionary conservation of *lmgA*, the 92 bp intercistronic sequence (ICS), *lmgB *and the 3' untranslated region (3'-UTR) of 12 Drosophilidae species were determined and compared. As Table 2 illustrates, the topology of the locus is maintained in these species, but the conservation of the different regions shows considerable variations. The amino acid sequence of LmgA shows the highest conservation (96% at minimum), while LmgB shows only 66% identity between the most widely diverged species. Though the ICS region varies in length (92-165 bps), together with the 3'-UTR, they still demonstrate a significant level of conservation among the Drosophila species analysed. Although the LmgB protein sequence is conserved in Drosophilidae, homologous sequences could not be found in other species.

**Table 2:** Bioinformatic analysis of the *lemming *locus in Drosophilidae species

Species	LmgA (aa%)	92 ICS(bp) %	LmgB (aa%)	3'-UTR (bp%)
*D. simulans*	100	(92) 97	145 aa, 90 143 aa, 99	92

*D. sechellia*	100	(92) 100	98	96

*D. yakuba*	100	(83) 88	98	91

*D. erecta*	100	(83) 86	97	91

*D. ananassae*	98	(96) 53	84	67

*D. pseudoobscura*	98	(111) 41	77	58

*D. persimilis*	98	(111) 41	76	59

*D. willistoni*	97	(157) 28	69	47

*D. mojavensis*	97	(165) 33	67	47

*D. virilis*	96	(162) 31	66	47

*D. grimshawi*	97	(150) 36	67	46

aa% and bp% represent amino acid and nucleic acid sequence similarities in different regions of the *lemming *locus from 11 species compared to *D. melanogaster *sequences.

ICS: intercistronic sequence; 3'-UTR: 3'-untranslated region

### The developmental transcription pattern of *lmgA *and *lmgB *is very similar

**Figure 6:**
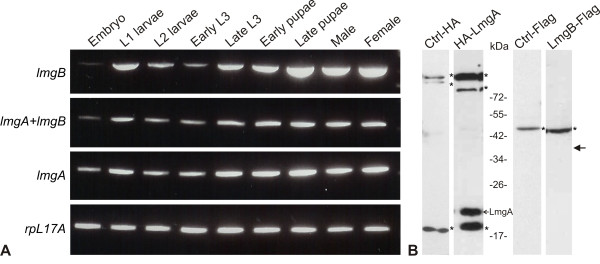
**Expression pattern of different *lmg *gene products**. Semi-quantitative RT-PCR was used to monitor the *lmg*-specific transcripts in total RNA samples from animals at different developmental stages (A). Western blots of total protein extracts from S2 cells transfected with the dicistronic *lmgAB *construct treated with anti-HA or anti- FLAG monoclonal antibodies (B). Even after overloading the anti-FLAG track, no FLAG-tagged LmgB could be detected. The arrow indicates the predicted molecular weight of LmgB. Bands labeled with asterisks are polypeptides nonspecifically recognized by anti-FLAG and anti-HA antibodies. Ctrl: Total protein extract from non-transfected cells treated with anti-FLAG or anti-HA antibodies.

Dicistronic genes are often products of a duplication event and related functionally. However, neither the nucleotide, nor the amino acid sequence of ORF1 and ORF2 showed any similarity. Nonetheless, the existence of mono- and dicistronic mRNAs raised the possibility of coordinated expression. To investigate this, total RNA was isolated from isogenized *w^1118 ^*embryos (0-12 hours), L1, L2, early L3, late L3 larvae, early pupae, late pupae, males and females. These samples were then used to analyze the expression pattern of monocistronic and dicistronic messages by semi-quantitative RT-PCR, using *lmgA, lmgB *and *lmgAB *(spanning the intercistronic region) specific primer pairs (Figure 2B and Materials and Methods). As Figure 6A shows, the expression patterns of these transcripts are very similar. In each case, lowest expression occurs in embryos, followed by a significant increase in first instar larvae. Low expression could be detected in second and early third instar larvae, and then it intensifies in late third instar larvae and early pupae. Late pupae show the highest expression level for these transcripts that then stays steady in male and female adults. This result implies that the dicistronic message is the main product of *lmg *transcription. If monocistronic *lmgA *or *lmgB *messages are present, their expression profile does not differ significantly from that of the dicistronic message.

### The *lmgB *ORF is not efficiently translated

Translation usually starts at the AUG codon of the upstream cistron, and it is suggested that translation of the second cistron of a dicistronic mRNA is initiated at an internal ribosome entry site (IRES) or by some kind of reinitiating processes [18]. Our *in silico *sequence analysis did not identify predicted IRES sites in the intercistronic region. In order to test the translation of the two ORFs, the dicistronic cDNA was cloned into a Gateway expression vector, in which a hemagglutinin (HA) tag was inserted in frame to the 5'-end of *lmgA*, and a Flag tag was ligated to the 3'-end of *lmgB*. Schneider 2 (S2) cells were transfected with this construct and, after propagation, total protein extract was prepared and analyzed by Western blots using anti-Flag and anti-HA monoclonal antibodies. As Figure 6B illustrates, while HA-tagged LmgA was produced, no LmgB-Flag protein could be detected. Thus, while the expression pattern of the dicistronic mRNA could be monitored throughout development and it was expressed in S2 cells, the translation of the downstream *lmgB *ORF could not be detected. However, it cannot be excluded that LmgB might be produced below detection sensitivity. It is also possible that by unfortunate coincidence, the LmgB-Flag protein migrates with a lower electrophoretic mobility than expected, resulting in comigration with the band recognized by anti-Flag nonspecifically (Figure 6B).

### Expression of *lmgA *alone rescues the lethal, morphological and mitotic phenotypes of the *lmg^138 ^*mutant

In order to determine if one or both ORFs were required for essential *lemming *functions, transgenic lines were established in a *lmg^138 ^*mutant background expressing either dicistronic (*lmgAB*) cDNA, or the *lmgA *or *lmgB *ORFs alone under control of the yeast transcription factor Gal4 [19]. The ability of these transgenes to rescue the lethal, morphological and mitotic phenotypes of *lmg^138 ^*was investigated. As Table 1 demonstrates, ubiquitous expression of the *lmgAB *or *lmgA *transgenes by the *da-Gal4 *driver was sufficient to rescue fully the lethal and mitotic phenotypes of *lmg^138^*, and to restore the normal development of imaginal discs and larval brain. In contrast, the ubiquitous expression of *lmgB *rescued neither the lethal nor the mitotic mutant phenotype in several independent transgenic lines, despite the fact that its expression at mRNA level could easily be detected by RT-PCR. These data demonstrate that the developmental and mitotic defects observed in *lmg *mutants reflect essential functions of *lmgA*. On the other hand, *lmgB *appears to be dispensable without any detectable effect.

### Heterologous expression of LmgA complements a *S. cerevisae apc11 *mutant

**Figure 7:**
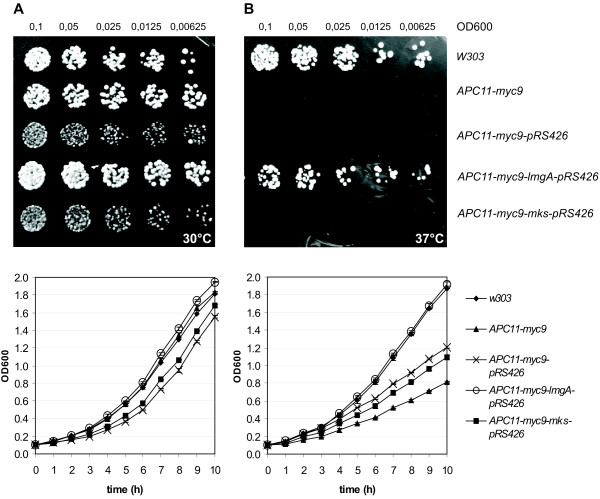
***lmgA *rescues the temperature-sensitive phenotype of a yeast *APC11-myc9 *null mutant**. W303 control, *APC11-myc9 *mutant and transformed *APC11-myc9 *mutant cells grow normally at 30°C on YEPD plates (A, upper panel) and in liquid medium (A, lower panel). However, at 37°C only the *APC11-myc9 *mutant cells transformed with the *lmgA*-pRS426 expression construct and control cells are capable of growing, both on YEPD plates (B, upper panel) and in liquid medium (B, lower panel). Cells were plated in linear dilution series; each drop containing half the number of cells than the previous one.

Since the predicted LmgA protein showed a very high degree of similarity to yeast and human Apc11 proteins, we wanted to test if LmgA was able to function as an Apc11 substitute in budding yeast cells defective in their endogenous APC11 function. For this, we introduced a *lmgA *expression construct under the control of the constitutively active alcohol dehydrogenase promoter into *APC11-myc9 *cells and tested for complementation of their temperature sensitive proliferation defect [11]. As Figure 7A and 7B show, *APC11-myc9 *cells grow well at 30°C but are unable to do so at 37°C, though the W303 control cells are quite capable of growing at the higher, restrictive temperature (Figure 7B). The ability of *APC11-myc9 *cells to grow at 37°C was restored following transformation with the pRS426-*lmgA *plasmid, which expresses LmgA constitutively. In parallel experiments, the *APC11-myc9 *cells failed to grow at the restrictive temperature when transformed with the empty vector (pRS426), or with the pRS426-*mks *plasmid expressing the Apc3 subunit of the Drosophila APC/C (Figure 7B). This result proves that the LmgA protein functionally complements the APC11 subunit in yeast and it suggests that the function of the Apc11 homologues is evolutionarily conserved.

### LmgA interacts with Apc2, and together, they bind Vihar

**Figure 8:**
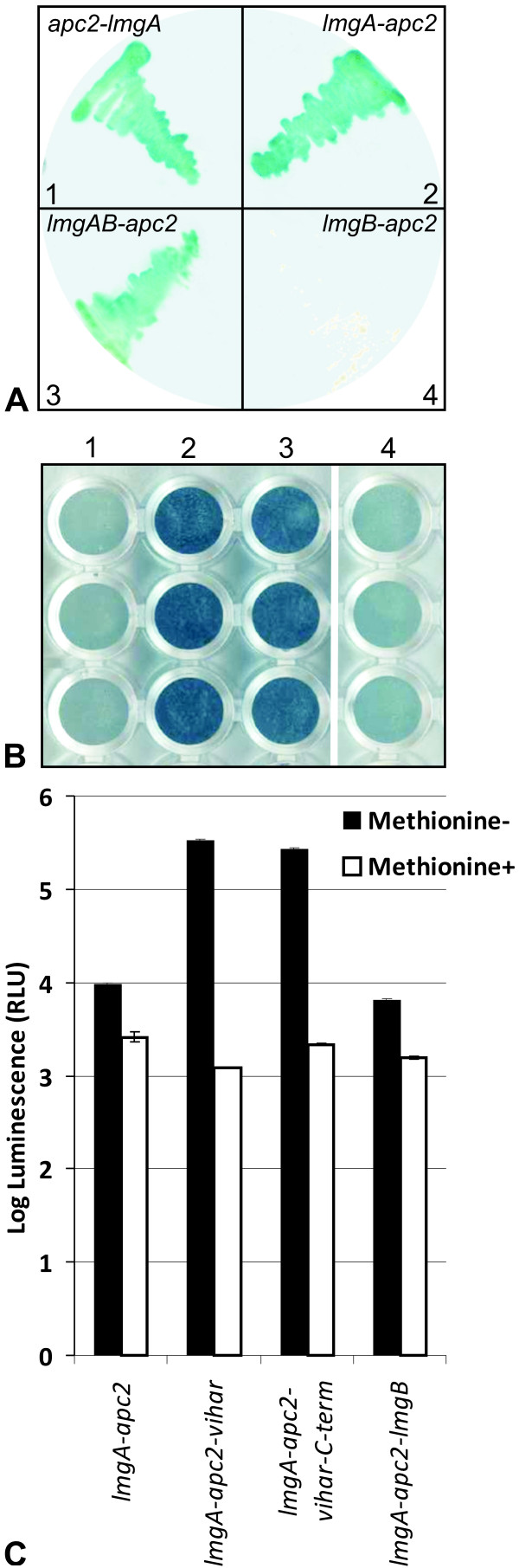
**LmgA interacts with APC2/Mr and they together interact with the E2 enzyme Vihar**. In yeast two-hybrid experiments, *S. cerevisiae *L40 strain was co-transformed with *apc2*-pGAD424 and l*mgA*-pBTM116 (A1), *apc2*-pBTM116 and l*mgA*-pGAD424 (A2), *apc2*-pBTM116 and *lmgAB*-pACT2 (A3), as well as with *apc2*-pBTM116 and *lmgB*-pGAD424 (A4) plasmids. Blue colonies indicate the interaction. In yeast three hybrid experiments, Tat7 cells were co-transformed with *lmgA*-pBTM116, *apc2*-pRS416 and pGAD424 (B1), *lmgA*-pBTM116, *apc2*-pRS416 and *vihar*-pGAD424 (B2), *lmgA*-pBTM116, *apc2*-pRS416 and *vihar*-C-term-pGAD424 (B3), and *lmgA*-pBTM116, *apc2*-pRS416 and *lmgB*-pGAD42 (B4) and PXG-assays were performed. Strong interactions are indicated by dark blue colorations in columns 2 and 3. The three rows represent parallel experiments. *Beta*-*Glo*^® ^assays were performed with the same transformants grown in liquid minimal medium, in the presence or absence of 1 mM methionine (C). Increased luminescence in *lmgA-Apc2-vihar *and *lmgA-Apc2-viharC-term *indicates interactions.

As it is known that the yeast and human APC2 protein interacts with APC11 and together they form the catalytic subcomplex of the APC/C [4], we examined if the Drosophila proteins interacted similarly. In a yeast two-hybrid (Y2H) assay, *apc2*-pBTM116 served as bait to screen a Drosophila embryo cDNA library cloned into the pACT2 vector (Clontech Laboratories, Inc., USA). Originally, this experiment served to identify Apc2 interacting proteins. A strong interaction was found with a clone that was purified and characterized. Sequence analysis showed that it contains two tandem ORFs in different frames matching the dicistronic mRNA of the *lemming *gene. To identify the region of the *lmg *cDNA required for *apc2*-pBTM116 interaction, the two segments corresponding to *lmgA *and *lmgB *were cloned into pGAD424 and examined in two-hybrid assays. Only LmgA showed strong interaction with Apc2, even if the fusions to the activation and binding domains were reversed (Figure 8A). A yeast two hybrid screen was also performed to find interacting partners for the hypothetical protein coded by *lmgB*. Using *lmgB*-pBTM116 as bait, no interacting clone could be identified in the cDNA library mentioned above.

It has been shown that Ubc4 and E2-C type E2s bind *in vitro *either to the Apc2 or Apc11 subunit of the catalytic subcomplex [7]. However, genetic analysis of E2 mutants in yeast and Drosophila suggests that, *in vivo*, the E2-C type enzymes are required for physiological APC/C function [20,21]. There is only one E2-C enzyme in Drosophila, encoded by the *vihar *gene [21]. Since the binding partner of Vihar and the nature of its interaction were not known, we used yeast two-hybrid assays to test for physical interactions between Vihar and either Apc2/Mr, or Apc11/LmgA. Though fusions of *vihar, Apc2/mr *and *Apc11/lmgA *coding sequences were made to all possible activation and binding domain combinations, no interactions could be detected. A possible explanation of this result is that the Apc2/Mr and Apc11/LmgA subunits jointly create a binding site for Vihar, so the three proteins could form a ternary complex. To assess this possibility, we used yeast three-hybrid assays in which interactions between *lmgA*-pBTM116, *vihar*-pGAD424 and the methionine-regulated *apc2*-pRS416 (see Materials and Methods) were analyzed. In this setting, interaction among these proteins was clearly observed (Figure 8B and 8C), indicating that both Apc2/Mr and Apc11/LmgA are required for Vihar binding.

## Discussion

The APC/C belongs to the cullin-RING family of multisubunit ubiquitin ligases. Previous studies of the budding yeast and human APC/C indicated that the cullin-related Apc2 and the RING-finger-containing Apc11 subunits together form the minimal ubiquitin ligase module [4,7]. We show in this paper that, in *Drosophila melanogaster*, the Apc11 subunit is encoded by the dicistronic *lemming *locus. The upstream ORF, *lmgA*, encodes a putative protein containing a RING-finger motif characteristic of known APC11 subunits and shows more than 80% sequence similarity with the APC11 subunit of the human APC/C. Since the Apc11 subunit is proposed to play a role in the catalytic center of the APC/C, mutations in *lmg *are expected to lead to loss of APC function, and therefore to aberrant cell cycle progression. The *lmg *mitotic phenotype presented in this paper is consistent with the Lmg protein being a subunit of the APC. The mitotic defects we observe in *lmg *larval neuroblasts, including metaphase-like arrest, chromosome overcondensation and polyploidy, in addition to widespread apoptosis of mitotically-active cells, are very similar to those reported for loss of other subunits of the Drosophila APC/C [12-14]. A role of LmgA in the APC/C is further supported by the elevated levels of cyclin A and B observed in *lmg *neuroblasts. Another line of supporting evidence comes from the synergistic genetic interaction between *lmgA *and *mr/Apc2 *and *lmgA *and *vihar *(Table 1B and 1C) and from the physical interactions among these proteins, since it is known that, in yeasts and vertebrates, these proteins form the catalytic module of the APC/C. These data, together with its ability to complement the mutant phenotype of yeast Apc11-deficient cells support the designation of *lmgA *as a true *Apc11 *orthologue.

The APC/C requires special E2 enzymes for activity and has been demonstrated to function with Ubc4/5 and E2-C type E2 enzymes *in vitro *[22,23]. Whereas in yeast and human cells the E2 enzymes bind to either Apc2 or Apc11, our data suggest that in Drosophila, both of these subunits are required for effective E2 binding. This could represent an architectural variation in the catalytic subcomplex of different APC/C ligases.

The dicistronic nature of the *lmg *locus is a notable but puzzling fact. Whereas the upstream *lmgA *ORF encodes the Apc11 subunit of the Drosophila APC/C, the existence and function of the predicted downstream *lmgB *ORF product remains unknown. We could not find any sequence or functional relationship between *lmgA *and *lmgB*, though such relationships are characteristic of many dicistronic genes [24]. Genomes of other species from Drosophilidae (especially in the melanogaster group) contain both these ORFs and the intercistronic sequence in a similar arrangement (data not shown). Moreover, the high evolutionary conservation of LmgA and LmgB and significant conservation of both ICS and 3'-UTR suggest functional relevance. However, we found that the putative LmgB is dispensable for the organism and lacks known protein motifs. In addition to this, no apparent LmgB interaction partners could be found in yeast two hybrid screen and LmgB could not be efficiently translated from the dicistronic mRNA in S2 cells. *lmgA *contains three in-frame AUG codons in addition to its initiating AUG codon. It has been shown for two Drosophila dicistronic transcripts, of the *stoned *and *snapin *loci, that such in-frame AUG codons effectively attenuate the translation of the second ORF [25]. However, the rationale for the dicistronic arrangement of the *lmgA *and *lmgB *cistrons and the function of the *lmgB *ORF remains obscure.

The mechanism by which loss of APC/C function leads to apoptosis is unknown but it may be significant that *lmg *mutant cells entered apoptosis directly, and rapidly, from arrested cells, without a return to the interphase state (data not shown). There is accumulating evidence that mitosis and apoptosis share components [26]. It has been suggested that apoptosis is a default pathway and proteins such as survivin are required to counteract this pathway during mitosis [27,28]. Cells treated with drugs which alter microtubule dynamics, such as paclitaxel (Taxol) also undergo mitotic arrest and enter apoptosis rapidly, and directly, from mitosis [29]. Since these drugs are thought to trigger the spindle assembly checkpoint which in turn acts by inhibiting the APC/C [30-32], it is possible that loss of APC/C activity is responsible for triggering apoptosis. Inactivation of the APC/C by cleavage of the CDC27 component by caspases has also been shown to occur during apoptosis triggered by Fas ligand in Jurkat cells, contributing to an increase in Cdk activity [33]. There have been several reports of increased Cdk activity during apoptosis [34-36], suggesting that these enzymes form part of the apoptotic pathway. Increased mitotic cyclin levels, and Cdk activity, may therefore play a role in apoptosis triggered by loss of APC/C function. Apoptosis, however, does not normally occur when cyclin levels are high at metaphase. This may be because of protective factors such as survivin [27,28]. A loss of protective activity during anaphase may allow cells to respond to abnormally high levels of Cdk activity and undergo apoptosis. Alternatively, if the APC/C itself plays a protective role, simultaneous loss of this protection and elevated Cdk levels would result in apoptosis.

The polyploid cells we observed in larval brain squashes may be cells that have escaped apoptosis, exited mitosis without cytokinesis, and then duplicated their chromosomes before re-entering mitosis again. If so, some cells can clearly repeat the process several times, as we observed cells that were highly polyploid. Furthermore, we did not observe any G2-arrested interphase larval abdominal histoblasts undergoing apoptosis (data not shown). This suggests that there is a phase, during mitosis, when cells are particularly sensitive to loss of *lmg *function and respond by undergoing apoptosis. This might be expected if loss of APC/C function is playing a relatively direct role in triggering apoptosis.

## Conclusions

The data presented in this paper demonstrate that the upstream member of a dicistronic gene, *lmgA *codes for the Apc11 subunit of the APC/C in a multicellular metazoan species, *Drosophila melanogaster*. Its genetic and physical interactions with Mr/Apc2 and the E2-C type ubiquitin-conjugating enzyme, Vihar, suggest that their ternary complex represents the same catalytic module of the APC/C that was identified in yeast and mammalian cells by functional means.

## Methods

### Yeast strains and techniques

Standard yeast media and yeast techniques were used [37]. *S. cerevisiae *L40 strain (*MATa ade2 his3Δ200 leu2-3,112 trp1Δ1 ura3::lexAop-lacZ LYS2::lexAop-HIS3*) was used for two hybrid, and the Tat7 strain (*MATa ura3-52 ade2 his3Δ200 leu2-3,112 trp1Δ1 ura3::lexAop-lacZ LYS2::lexAop-HIS3*, generously provided by Jacques Camonis) for three hybrid analysis. W303 (*MATa ade2-1 tpr1-1 can1-100 leu2-3, 112 his3-11,15 ura3*) and an *APC11-myc9 *strain (*MATa APC11myc9-TPR1 ade2-1 tpr1-1 can1-100 leu2-3, 112 his3-11,15 ura3*, kindly provided by Wolfgang Zachariae) were used for the heterologous complementation experiments.

### Yeast two and three hybrid experiments

A Drosophila embryonic cDNA library cloned into the pACT vector (Clontech Laboratories, Inc., USA) was screened using *apc2*-pBTM116 as bait as described by the manufacturer (Clontech Laboratories, Inc., USA). The *lmgA *and *lmgB *sequences were cloned into pGAD424 and pACT2 respectively, and then co-transformed with *apc2*-pBTM116 into L40 strains to confirm their interaction. The cDNA library was also screened with the *lmgB*-pBTM116 clone as bait. Yeast three hybrid analyses were performed using *lmgA*-pBTM116, *apc2*-pRS416 (kindly provided by Jacques Camonis) and *vihar*-pGAD424 which were co-transformed into the Tat7 strain. His^+ ^colonies were selected and tested for their β-galactosidase activity.

### β-galactosidase assay

Colony-lift filter assays were performed as described in the Yeast Protocols Handbook (Clontech Laboratories, Inc., USA). For quantification of β-galactosidase activity, single colonies were grown until mid-log phase in liquid minimal medium containing 1 mM 3-Amino-1,2,4-triazole, and in the presence or absence of 1 mM methionine. Liquid cultures were used for Pellet X-Gal (PXG) assay [38] and for *Beta*-*Glo*^® ^*Assay *according to the manufacturer's protocol (Promega Corporation, USA).

### Yeast heterologous complementation test

The *lmgA *cistron was cloned into the pRS426 yeast expression vector (kindly provided by Ildikó Unk). As a control the *mks/Apc3 *gene was also cloned into pRS426. *APC11-myc9 *temperature sensitive mutant cells [11] were transformed with *lmgA*-pRS426, *mks*-pRS426 and empty pRS426 plasmids. Single Ura^+ ^colonies were selected on minimal medium then inoculated into 3 ml YEPD (BIO 101, Inc., Canada) media and incubated overnight at 30°C in a water bath. Overnight cultures were diluted to OD_600 _= 0.1, and divided into 6 glass tubes, 3 ml each. 3 tubes were incubated at 30°C and 3 tubes at 37°C in water baths for 10 hours. The W303 strain was used as a control. Optical density was measured every hour using a WPA Biowawe CO8000 Cell Density Meter. Data were analyzed and densitometric growth curves were made using Microsoft Office Excel™. For the colony forming dilution, single colonies were inoculated and grown overnight at 30°C. Cultures were diluted to OD_600 _= 0.1 and then two-fold serially diluted five times. 5 μl of each dilution was spotted onto YEPD plates which were incubated at 30°C and 37°C for 1 day.

### Drosophila stocks and genetic techniques

Fly stocks were reared on standard yeast/dextrose medium at 25°C. Stocks were obtained from the Bloomington Drosophila Stock Center. In all experiments a *w^1118 ^*isogenic stock was used as the control. All genetic markers used are described in Flybase http://flybase.org.

To determine the lethal phase, chromosomes carrying the mutations were balanced over *CyO, actGFP *or *TM6C, Tb, Sb *chromosomes. From each line 20-30 pairs of flies were placed into chambers on agar plates containing yeast extract. 400-600 first instar homozygous and heterozygous larvae were collected and put into vials, 50-50 each. Metamorphosis was staged according to Bainbridge and Bownes [15].

### P-element remobilization

Originally, both the P{PZ}03424 and P{EPgy2}EY11317 mutant stocks carried second site mutations which were removed by recombination before experimental use. Imprecise excisions of the P{PZ}03424 and P{EPgy2}EY11317 elements were generated by crossing to flies carrying the Δ2-3 transposase. Chromosomes which had lost the *ry^+ ^*or *w^+ ^*genes in the P element were selected and balanced over *CyO, actGFP*. For deletion mutant screening, genomic DNA was extracted from candidate lines as by Gloor et al [39] and PCR was performed using the *lmg *specific 5'-CTCCCGCCAAGGATCGATATCTTT-3' and 5'-TATGTTGGGGATGTTGGTGTGAATG-3' primers. The P{PZ}03424 remobilization generated the *lmg^J023 ^*allele that carries a 1543 bp deletion, while the P{EPgy2}EY11317 imprecise excision yielded two independent lines carrying deletions in the *lmg *gene. In this study we used the *lmg^138 ^*null mutant which carries an 1120 bp deletion downstream from the P-element insertion site.

### Rescue of the *lmg *mutant phenotype

Dicistronic *lmgAB*, and the *lmgA *and *lmgB *cistrons alone were cloned into the pUAST vector. The resulting plasmids were sequenced and injected into *w^1118 ^*embryos according to standard protocols [40]. Homozygous transgenic flies carrying one of the *lmgA*-pUAST, *lmgB*-pUAST and *lmgAB*-pUAST constructs on the third chromosome were crossed to *lmg *mutants and *w/w; lmg^X^/CyO, actGFP; Y*-pUAST lines were established, in which X represent one of the *lmg^03424^, lmg^Ey11317^, lmg^J023^, lmg^138 ^*alleles and Y stands for the different *lmg*-pUAST constructs in all combinations. To express *lmg *in these constructs, *w/w; lmg^X^/CyO, actGFP; Y*-pUAST flies were crossed to *w/w; lmg^X^/CyO, actGFP; da-GAL4/da-GAL4 *flies and resulting progeny of *w/w; lmg/lmg; *pUAST/*da-GAL4 *were tested for lethal and mitotic phenotypes.

### Semi-quantitative RT -PCR

Total RNA was isolated using a Tri Reagent extraction kit (Sigma-Aldrich, USA). RNA samples were treated with RQ1 RNase-Free DNase (Promega Corporation, USA). Reverse transcription was carried out using a Fermentas cDNA synthesis kit using 5 μg RNA and random hexamer primers. cDNA amounts were normalized in 20 cycle PCR using *rpL17A *primers (*rpL17A *upper, 5'-GTGATGAACTGTGCCGACAA-3'; *rpL17A *lower, 5'-CCTTCATTTCGCCCTTGTTG-3'). PCR products were separated by electrophoresis in a 1.2% agarose gel. To analyze the expression pattern of the *lmg *locus during development, semi-quantitative RT-PCRs were performed. Total RNA was isolated from 50 mg *w^1118 ^*0-12 hours embryos, L1, L2, early L3, late L3 larvae, early pupae, late pupae, males and females. The following primers were used in 25 cycle PCRs: ORF1 RT primers: 5'-GCAAAGCGGCGACAAAC-3' upstream and 5'-CAGCTCTGGCGGCACAT-3' downstream; ORF2 RT primers: 5'-AATGATGGAGAACAGCAGCAACGAT-3' upstream and 5-TATGTTGGGGATGTTGGTGTGAATG-3' downstream; Dicistronic RT primers: 5'-CCGCTGGTGTGGGGTGTATG-3' upstream and 5'-TTTGGCAGTGGCGGCAGAC-3' downstream.

### Cytological analysis and Immunohistochemistry

Orcein staining of larval brain preparations was carried out and analyzed as described previously [14]. Preparations were examined under an Olympus BX51 microscope using phase contrast. Photos were taken by a DP70 digital color camera. Mitotic index was determined as the number of cells in mitosis in an optical field. Apoptotic index was defined as the number of rounded cells with picnotic nuclei in an optical field. Apoptosis was also analyzed by acridine orange staining of dissected brains and wing imaginal discs. Heads of third instar larvae were removed in PBS and transferred into a drop of 1.6 μg/μl acridine orange solution (C.I. 46005 Molar Chemicals Ltd) for five minutes in the dark, then rinsed in PBS. Brains and wing imaginal discs were then dissected and transferred to a drop of PBS on a microscope slide. Before transferring, two cellotape cushions were made on the slide to prevent excess compression. Preparations were covered with a cover-slip, sealed with nail-polish and examined under an Olympus BX51 upright microscope, or with an Olympus FV 1000 confocal microscope.

For immunohistochemistry, brains were dissected from *w^1118 ^*and *lmg^J023 ^*wandering third instar larvae. Immunostaing preparations, primary and secondary antibodies and image analysis were performed as described in Pál et al. [14].

### RACE Experiments

Rapid Amplification of cDNA ends was performed using a First-Choice™ RLM-RACE kit (Ambion, USA) according to the protocol provided by the manufacturer. Total RNA was isolated from 50 mg 0-12 hour embryos with Tri Reagent (Sigma-Aldrich, USA). To obtain the 5' end of mRNAs gene specific primers were designed to ORF1 (5'-TCCGGGCAGGTGCTCTCG-3' inner and 5'-CAGCTCTGGCGGCACAT-3'outer) and ORF2 (5'-TTTGGCAGTGGCGGCAGAC-3' inner and 5'-CTGGAAGCGCGACTGTGC-3' outer). To obtain the sequence of the 3' end of the ORF1 mRNA, PCR was carried out with specific primers for ORF1 (5'-GCAAAGCGGCGACAAAC-3' outer and 5'-CCGCTGGTGTGGGGTGTATG-3' inner). All PCR products were cloned into pTZ57R (Fermentas, Vilnius, Lithuania) and sequenced.

### Epitope construct and Western Blots

To test the expression of the two *lmg *ORFs, an N-terminal Hemagglutinin tag was attached to ORF1, and a C-terminal Flag tag was made for ORF2. The 5'-TCGAGATTACAAGGACGATGACAAGTAG-3' and 5'-CTACTTGTCATCGTCGTCCTTGTAATC-3' oligonucleotides were ligated into the *Eco*RV-*Xho*I sites of pENTR1A, and transformed into DB3.1 competent cells. The *lmgAB *sequence was then ligated into the pENTR1A-Flag tagged construct. The pENTR1A-*lmgAB*-Flag was recombined into the pAHW destination vector, which contains three HA epitope tags 5' in its Gateway cassette, using Gateway LR clonase II Enzyme Mix (Invitrogen Corporation, USA). Recombinant clones were selected by ampicillin resistance and sequenced before transfection.

Schneider 2 (S2) cells were transfected with the FLAG-*lmgAB*-HA plasmid using Cellfectin (Invitrogen Corporation, USA) in serum-free S2 cell medium. Total protein was extracted from transfected cells using standard protocols [41] and analyzed by Western blots using anti-Flag and anti-HA monoclonal antibodies.

## Competing interests

The authors declare that they have no competing interests.

## Authors' contributions

ON carried out the genetic, molecular and cytological characterizations of *lmg *mutants and isolated the *lmg *null allele. MP participated in the yeast two- and three-hybrid experiments and in immunohistochemistry. AU carried out the construction and expression of tagged protein expression constructs. CAMS carried out analysis of the *lmg^03424 ^*and *lmg^JO23 ^*mutants and initiated molecular analysis of the *lmg *locus. IB set up the yeast two-hybrid experiments and critically revised the manuscript. ADS identified the *lmg^03424 ^*allele and helped to draft the manuscript. PD planned and coordinated the experimental work and drafted the manuscript. All authors read and approved the final manuscript.
